# Nutrition and Calcitonin Gene Related Peptide (CGRP) in Migraine

**DOI:** 10.3390/nu15020289

**Published:** 2023-01-06

**Authors:** Michal Fila, Jan Chojnacki, Piotr Sobczuk, Cezary Chojnacki, Janusz Blasiak

**Affiliations:** 1 Department of Developmental Neurology and Epileptology, Polish Mother’s Memorial Hospital Research Institute, 93-338 Lodz, Poland; 2 Department of Clinical Nutrition and Gastroenterological Diagnostics, Medical University of Lodz, 90-647 Lodz, Poland; 3 Emergency Medicine and Disaster Medicine Department, Medical University of Lodz, 92-209 Lodz, Poland; 4 Department of Orthopaedics and Traumatology, Polish Mothers’ Memorial Hospital Research Institute, Rzgowska 281, 93-338 Lodz, Poland; 5 Department of Molecular Genetics, Faculty of Biology and Environmental Protection, University of Lodz, 90-236 Lodz, Poland

**Keywords:** dietary nutrients, calcitonin gene-related peptide, CGRP, migraine, anti-CGRP therapy, drugs-diet interaction, food intake control

## Abstract

Targeting calcitonin gene-related peptide (CGRP) and its receptor by antibodies and antagonists was a breakthrough in migraine prevention and treatment. However, not all migraine patients respond to CGRP-based therapy and a fraction of those who respond complain of aliments mainly in the gastrointestinal tract. In addition, CGRP and migraine are associated with obesity and metabolic diseases, including diabetes. Therefore, CGRP may play an important role in the functioning of the gut-brain-microflora axis. CGRP secretion may be modulated by dietary compounds associated with the disruption of calcium signaling and upregulation of mitogen-activated kinase phosphatases 1 and 3. CGRP may display anorexigenic properties through induction of anorexigenic neuropeptides, such as cholecystokinin and/or inhibit orexigenic neuropeptides, such as neuropeptide Y and melanin-concentrating hormone CH, resulting in the suppression of food intake, functionally coupled to the activation of the hypothalamic 3′,5′-cyclic adenosine monophosphate. The anorexigenic action of CGRP observed in animal studies may reflect its general potential to control appetite/satiety or general food intake. Therefore, dietary nutrients may modulate CGRP, and CGRP may modulate their intake. Therefore, anti-CGRP therapy should consider this mutual dependence to increase the efficacy of the therapy and reduce its unwanted side effects. This narrative review presents information on molecular aspects of the interaction between dietary nutrients and CGRP and their reported and prospective use to improve anti-CGRP therapy in migraine.

## 1. Introduction

Migraine is a common disabling disorder affecting mainly individuals in productive age and consequently resulting in a high burden for societies. Despite its high prevalence and serious consequences, it is still an undertreated disorder. The main reason for this may be poor knowledge of molecular mechanisms underlying migraine induction and progression.

Calcitonin gene-related peptide (CGRP) is a neuropeptide, which, along with its receptor, is involved in pain transmission and released from trigeminal termini during migraine attacks [1]. However, where and how CGRP works in migraine is not fully known. The development of monoclonal antibodies against CGRP and its receptor for migraine prophylaxis and antagonists of the CGRP receptor (gepants) for the treatment of acute migraine attacks and their prevention was a breakthrough in migraine treatment (reviewed in [2]). However, some questions about the long-term safety of these drugs and the resistance of certain patients remain. Furthermore, the question of safety and efficacy in children and older adults is still open. Studies on the pharmacokinetics and pharmacodynamics of these drugs may stimulate progress in answering these questions, but environmental conditions and the patient’s lifestyle may contribute to all aspects of drug action. The diet is an important element of such environmental/lifestyle influences.

Dietary nutrients may modulate the action of many therapeutic drugs. Appropriate diet and hydration are essential in ameliorating the side effects of anticancer and antiviral drugs [3]. This may especially be important in migraine and anti-CGRP therapy, as many dietary compounds are migraine triggers. “Food to add and avoid” is recommended in many kinds of therapy. Moreover, body composition and weight, dependent on the diet, influence pharmacokinetics parameters of the anti-CGRP pathway monoclonal antibodies [4]. Furthermore, CGRP mediates central anorexigenic effects and is also found, along with its receptor, in the gastrointestinal (GI) mucosa [5,6]. Therefore, diet components may directly interact with CGRP and its receptors, but on the other hand, CGRP may modulate appetite, a key factor in food intake [7].

In this narrative review, we present and update information on molecular aspects of the interaction between dietary nutrients and CGRP, their reported and putative clinical implications and perspectives on improving anti-CGRP therapy in migraine with appropriate nutrition. We also discuss some nutritional issues of metabolic disorders, including obesity and diabetes, that may associate with migraine and the CGRP system. Some basic information on CGRP and migraine pathogenesis is also provided.

## 2. Migraine as a Diet-Sensitive Disease

Migraine is a neurological disorder involving sensory abnormalities occurring prior to, during and following a headache [8]. Historically, migraine was perceived as a vascular disease, but nowadays, it is generally accepted that changes in vasculatures in the form of vasodilation are neither necessary or not sufficient to induce migraine attack [2]. However, such changes can be targeted in some antimigraine treatments. Today, migraine is considered a disorder with a neurogenic rather than vascular basis, but neurogenic effects may include cerebral and meningeal arterial vasodilation [9]. Blood vessels contain cells that release intermediates acting on the brain neurons and contributing to migraine pathogenesis. On the other hand, neurons release signals that can be received by blood vessel cells. Therefore, the vascular basis of migraine fits into its neurogenic counterpart creating the neurovascular concept of migraine (reviewed in [10]). In addition, neurogenic inflammation may also directly contribute to migraine pathogenesis [9].

In fact, the pathogenesis of migraine is poorly known, and many factors, both genetic/epigenetic and environmental/lifestyle, can be involved (reviewed in [11]). Migraine is considered a threshold disease induced by a brain-related trigger, and some dietary nutrients are considered as such triggers (reviewed in [8,12]). However, some other nutrients, including nutraceuticals, may play a role in migraine prevention and prophylaxis [13,14,15,16]. Although many papers suggest an important role of diet modifications in migraine prevention and treatment, these suggestions lack a strong evidence base (reviewed in [17]).

Comprehensive diets regulate the right proportion between the core components of foods, including minerals, vitamins, proteins, carbohydrates and fats. However, some individuals report decreases in the frequency of headache attacks and/or their intensity after modifications to such a comprehensive diet [12]. In clinical practice in migraine, diet modification has the form of an elimination diet, which includes a long list of dietary substances considered as potential migraine triggers, such as chocolate, eggs, dairy products, alcoholic and sweetened beverages, citrus fruits and others [18,19]. These foods do not have too much in common, so the question about the involvement of appetite pathways in migraine induction is justified [20].

Several kinds of diet were postulated to prevent migraine or mitigate its effects (Table 1), but there is no strong evidence supporting these postulates (reviewed in [12,17,21]). Furthermore, some conflicting results were also obtained; for example, a low-calorie diet may result in a deficiency of electrolytes that leads to headaches [22]. It was shown in a cross-sectional study that women who adhered to a “low-quality diet” featuring increased intake of processed meat, nuts, sweetened beverages, fast food, and snacks had a higher frequency of headache attacks than women who had a “healthy diet” (increased intake of fruits, fish, vegetables, and legumes) [23].

Not only the diet content but also the meal schedule was associated with migraine. A higher prevalence of migraine was shown in individuals who had not a regular diet and regular diet schedule and who had fewer than three meals per day [17]. Diet composition and its regime are associated with weight management, and obesity is a risk factor for migraine [40,41].

Nutraceuticals, food or dietary supplements exerting beneficial health effects were studied in migraine prevention and therapy (reviewed in [16]). They are magnesium, coenzyme Q10, feverfew, riboflavin, phycocyanin, vitamin D and others. These studies do not always relate nutraceuticals action to CGRP concentration and suggest that such non-pharmacological treatment may be especially useful, but with modest efficacy, in headaches prevention in adolescents, pregnant or breastfeeding women, the elderly with complex drug therapy, patients who are inadvisable to pharmacological therapies. These non-pharmacological therapies are usually well-tolerated and safe. It was observed that women with migraine had lower plasma levels of Mg, Ca, Cu, and Zn than controls, but their dietary intake of Mg, Cu, and Fe was lower than recommended [42]. Therefore, patients with migraine may have lower plasma levels of minerals, and dietary intervention to ensure adequate mineral intake may be beneficial for them.

It is not the purpose of this work to present general information on associations between dietary nutrients and migraine. For this work, such information is important in the context of the CGRP system and CGRP-targeted antimigraine therapy. It should be underlined that migraine may be a diet-sensitive disease, and this should be considered when antimigraine treatment is to be modified by dietary compounds.

## 3. CGRP and Its Role in Migraine Pathogenesis and Treatment

Human CGRP is encoded by the calcitonin-related polypeptide alpha (*CALCA*) gene located at 11p5a. Alternative processing of the *CALCA* gene produces calcitonin (CT) and CGRP-alpha (CGRP1), one of the two isoforms of CGRP [43,44]. The other isoform, CGRP-beta (CGRP2), is encoded by the *CALCB* gene, but CGRP-alpha is a predominant CGRP form in the trigeminal ganglia and will be further referred to as CGRP unless otherwise stated. CGRP is a 37-aa neuropeptide involved in many phenomena and effects, including pain transmission and migraine [45,46]. CGRP preferentially binds calcitonin receptor-like receptor (CLR), receptor activity modifying protein 1 (RAMP1) and a small receptor component protein (RCP) [47]. CGRP, along with the CLR-RAMP1 complex, was found centrally in the trigeminovascular system [48].

It was observed that migraine patients had an elevated level of CGRP in physiological fluids during headache attacks, and migraine-like headaches were induced after the infusion of CGRP (reviewed in [49]). However, these associations did not provide information on the exact mechanism of CGRP’s involvement in migraine pathogenesis. In the central nervous system (CNS), CGRP is stored in vesicles in the sensory nerve terminals and trigeminal axons discharge CGRP into meninges blood vessels, inducing vasodilation and activation of trigeminal neurons [8,50]. CGRP also targets other CNS and peripheral neurons, glial cells and dural mast cells, triggering a cascade of effects related to neuroinflammation, allodynia, and other sensory effects typical for migraine [9]. Clinically, CGRP is claimed to be involved in migraine-like symptoms: light aversion (photophobia), decreased movement, spontaneous pain resulting in a grimace and evoked pain (mechanical allodynia) [2].

The treatment of migraine remains challenging, and a possible causative role of CGRP in migraine stimulated studies on its therapeutic utilization in this disease, which resulted in the development of the first migraine-specific preventive treatment drugs. Three classes of therapeutics targeting the CGRP system were approved for both chronic and episodic migraine treatment and prevention. These are (1) monoclonal antibodies against CGRP; (2) a monoclonal antibody targeting the canonical CGRP receptor, and (3) small molecular weight antagonists of CGRP receptors (gepants) (reviewed in [51]). These drugs revolutionized migraine therapy, but it is too early to draw definite conclusions about their efficacy and safety in long-term use, especially in some subpopulations and for today, we can only say that added value may be more promising efficacy over side effects [52]. The relatively high price of these drugs may also restrict their use, causing a delay in their full assessment. Therefore, studies to increase their efficacy and reduce unwanted side effects are justified.

**Figure 1:**
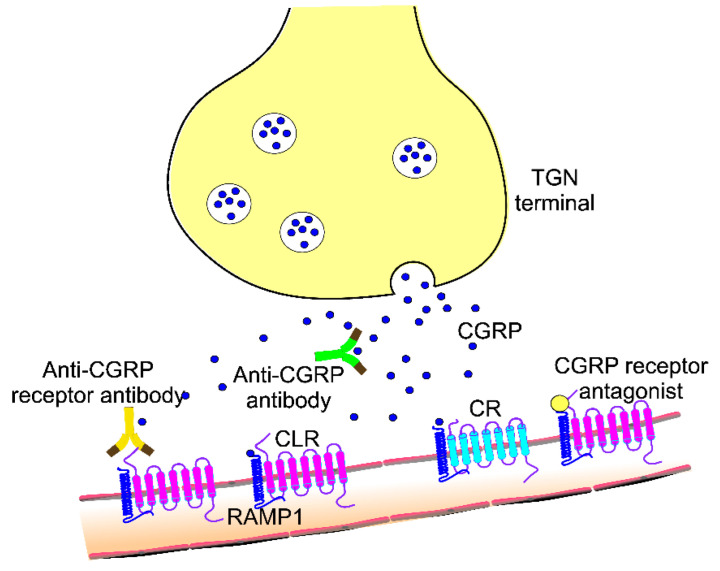
Pharmacodynamics of binding calcitonin gene-related peptide (CGRP) and its receptors by monoclonal antibodies and receptor antagonists (gepants) in the trigeminovasculature. CGRP is released from free endings of trigeminal nerve (TGN) afferents and can be blocked by its antibodies, preventing its binding with receptors and possible internalization, leading to dural vasodilation and neurogenic inflammation and positive feedback leading to peripheral sensitization. CGRP may bind its canonical receptor consisting of a G protein-coupled receptor (not presented here), calcitonin-like receptor (CLR) and an accessory protein, receptor activity-modifying protein 1 (RAMP1). A second receptor, the AMY-1 receptor, consisting of a calcitonin receptor (CR) and RAMP1, may also be targeted by CGRP. Antibodies against the CGRP receptor and its antagonists block CGRP binding. CGRP may cause cerebral vasodilation leading to central sensitization, but the exact mechanism of action antibodies against CGRP and its receptor is poorly known as these antibodies do not appreciably cross the blood-brain barrier.

The canonical CGRP receptor is a complex of a G protein-coupled receptor (GPCR), calcitonin-like receptor (CLR) and receptor activity-modifying protein 1 (RAMP1), but the second receptor, AMY-1, consisting of calcitonin receptor (CR) and RAMP1 may also be bound by CGRP [53] (Figure 1). CGRP competes with amylin and its analog pramlintide to bind AMY-1. Receptor internalization induced by CGRP binding may be crucial for the pro-migraine action of CGRP, and antibodies against CGRP receptors prevent this effect. CGRP receptor may also show internalization upon binding its antagonists (gepants). Yet, the AMY-1 receptor shows little internalization. However, these modes of internalization and their role in migraine pathogenesis are still discussed [54,55]. In general, the physiological role of constitutive receptor internalization of GPCRs is unclear and requires further research. However, some results suggest that certain GPCRs, including the CGRP receptor, can send signals from intracellular compartments [46]. Therefore, constitutive internalization of CGRP receptors can support sustained cellular responses after temporary ligand stimulation. Emerging evidence suggests an important potential of AMY-1 receptor in migraine therapy, as initially considered as insensitive to CGRP receptor antagonists, now AMY-1 is thought to contribute to anti-CGRP drugs action [56].

Although the precise mechanism of the involvement of CGRP in migraine is still not fully understood, its vasodilatory effect on cranial arteries as a link to migraine was established [57]. As suggested by Andrew Russo, the neurovascular processes, central in migraine pathogenesis, could be triggered in both the meninges and CNS, and they may proceed in both the neural-to-vascular and vascular-to-neural directions [2]. Perivascular action of CGRP involves a cascade of signals from the trigeminal vasculature to the thalamus and eventually the cortex. Anti-CGRP antibodies block some, but not all, CGRP molecules, preventing their binding with their receptors (Figure 1). A canonical CGRP receptor can be blocked by antibodies against it, but CGRP may still signal at the AMY-1 receptor. Therefore, in the case of antibody action, AMY-1 may present compensatory mechanisms for the suppression of CGRP signaling. Canonical CGRP receptors can also be blocked by their antagonists, which represent a chemically heterogeneous group of agents. One of them, eranumab, was reported to block both the canonical CGRP receptor and the AMY-1 receptor, likely due to its structure that may fit structurally similar clefts of the CLR/RAMP1 and CR/RAMP1 complexes and the cross-reactivity of gepants to both them [55].

Interaction between drugs and dietary nutrients may result in changes in their metabolism and transport [58]. However, direct modulation of drug action by food is also possible. As CGRP-targeting drugs are relatively new, there is not too much research on the influence of dietary nutrients on their efficacy and side effects. However, the importance of this problem is evident as dietary recommendations are issued along with the prescription of many drugs. Moreover, such recommendations may involve lowering the body mass as obesity, and metabolic impairments are associated with many disorders and can modulate the action of some drugs. On the other hand, food-drug interaction is usually complex and can be modulated by many factors that may differently affect both drugs and foods.

## 4. Modulation of CGRP by Dietary Nutrients

Some studies show that certain kinds of diet affect circulating CGRP levels, suggesting that certain components of the diet may influence migraine outcomes (reviewed in [59]). Secretion of CGRP may be triggered by the disruption of calcium signaling, and this mechanism is claimed to be involved in the modulation of CGRP secretion by food components [60]. A decreased release of CGRP from the neuroendocrine CA77 cells after their treatment with ginger and grape pomace extracts was observed [61]. In addition, S-petasin, a suspected active constituent of butterbur extract and a migraine prophylactic dietary supplement, also decreased CGRP release. However, S-petasin and ginger extract inhibited calcium influx, but grape pomace had no effect. It was concluded that grape pomace, ginger extracts, and S-petasin might have an anti-migraine potential through their anti-inflammatory actions, which might be underlined by different mechanisms.

Grape seed extract was shown to suppress basal expression of CGRP in spinal neurons in rats [62]. This effect was associated with an increase in the expression of mitogen-activated kinase phosphatase-1 (MKP-1).

Cocoa bean preparations have a long history of health-related beneficial effects [63]. They display antioxidant activity, which can be attributed, at least in part, to flavonoids, including catechin, epicatechin, and procyanidins, whose tricyclic structure enables scavenging reactive oxygen species, chelating Fe^2+^ and Cu^+^, and modulating pro- and antioxidant enzymes.

**Figure 2:**
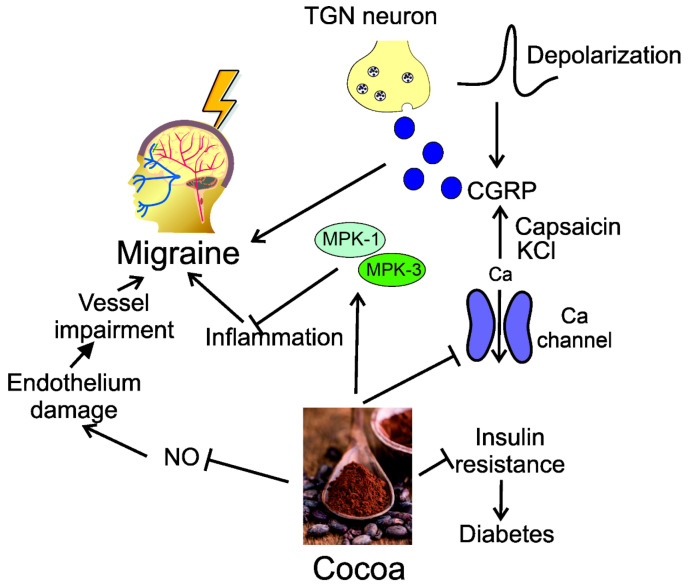
Cocoa is an example of a natural dietary nutrient that may exert beneficial effects against migraine, which may be partly underlined by its interaction with calcitonin-related gene peptide (CGRP) and represents mechanisms that may underline the action of other dietary nutrients on migraine and the CGRP systems. CGRP may be released after the depolarization of trigeminal nerve (TGN) neurons with the involvement of calcium signaling. Cocoa may disrupt calcium channels resulting in changing CGRP release, mediated by capsaicin and KCl. Cocoa may directly inhibit NO production, decreasing vessel impairment underlined by NO-induced endothelial damage. Coca may upregulate mitogen-activated kinase phosphatase-1 (MKP-1) and -3, which exert an anti-inflammatory effect, which is important in migraine prevention. Cocoa is also reported to break insulin resistance preventing diabetes, whose connection with migraine is discussed further in the main text.

On the other hand, the epicatechin content of cocoa is associated with its beneficial effects on vascular endothelium through its impact on both acute and chronic upregulation of nitric oxide (NO) production [64]. Cocoa polyphenols also show anti-inflammatory and pro-immune properties that can further contribute to their protective effects in vessels [63]. Therefore, cocoa may influence important pathways in migraine pathogenesis. In addition, cocoa's antioxidant properties may be involved in breaking insulin resistance and reducing the risk of diabetes [65]. It was shown that the treatment of rat trigeminal ganglia cultures with a cocoa extract reduced CGRP release, which was increased by depolarizing stimuli [66]. Cocoa also blocked the KCl- and capsaicin-stimulated increases in intracellular calcium. Those results showed that cocoa extract might repress stimulated CGRP release by a mechanism involving inhibition of calcium channels and suggest that diets rich in cocoa may suppress trigeminal sensory nerve activation. Subsequent studies showed that cocoa extract prevented inflammatory response in trigeminal ganglion neurons, which can be underlined by the upregulation of MKP-1 and MKP-3 [67] (Figure 2). These studies confirm the important role of MKP-1 in CGRP response to the intake of dietary nutrients.

Lower levels of CGRP in serum during episodic migraine were observed after dietary supplementation with vitamin D (2000 IU per day) as compared with individuals who received a placebo [68]. This effect was associated with some beneficiary changes in migraine characteristics, such as a decrease in the number of headache days per month and migraine-related disability score.

Beneficial effects in migraine were observed for some synthetic formulations of natural compounds—in general, they are moderately effective and generally safe (reviewed in [69]). Ginkgolide B, melatonin, histamine, oxytocin, various ribosomal peptide toxins, kavalactones, devil’s claw-derived compounds, salvinorin A and petasin, are among those agents that may be considered to support migraine prevention and treatment (reviewed in [69]). For example, curcumin, a natural herb product reported to exert many beneficial health effects, including anti-inflammatory action, was shown to ameliorate migraine syndromes and lower CGRP levels [70]. Curcumin 8-week supplementation in migraine patients was associated with a reduction in CGRP, IL-6, severity, and duration of headaches compared to controls. It was concluded that curcumin supplementation improved the inflammatory markers and clinical characteristics of migraine that could be attributed to its anti-inflammatory properties.

Cinnamon is reported to display anti-inflammatory and neuroprotective properties and modulate some hippocampal functions [71]. It was observed that serum concentrations of IL-6 and NO, as well as the frequency, severity and duration of migraine attacks, were reduced in migraine patients who received cinnamon as compared with the placebo group, but CGRP levels did not change in either group [72]. These results are somehow surprising—cinnamon had an apparent, molecularly documented anti-migraine effect, but it seemed not to affect the CGRP level. Therefore, further studies on the role of this agent in migraine and its interaction with the CGRP system are needed.

## 5. Anorexigenic Potential of CGRP and Its Involvement in the Appetite and Satiety Control

The regulation of appetite and feeding behavior involves both central and peripheral neurohormonal mechanisms that are not completely known [73]. In CNS, appetite and food intake are regulated in the hypothalamus and brainstem that send neuropeptides and feedback signals from the periphery, first of all, the gastrointestinal (GI) tract [74]. CGRP near CLR-RAMP1 and AMY-1 receptors was found in the human stomach, ileum and colon [75].

It was shown in several studies that CGRP exhibited central anorexigenic effects in animals, but these studies may not reflect general CGRP properties in appetite/satiety and food intake regulation [76,77]. It was observed that CGRP suppressed food intake when administrated through intracerebroventricular injection in rats deprived for 24 h of food intake [5]. Moreover, lower doses of CGRP decreased nocturnal food intake. Centrally administrated CGRP was more effective than its peripheral counterpart, but it did not change circulating glucose levels. Similar results were obtained in chicks, in which central or intraperitoneal injection of CGRP independently reduced food and water intake [78]. It was observed in that study that central injection of CGRP caused increased c-Fos (Fos proto-oncogene, AP-1 transcription factor subunit) immunoreactivity in the arcuate nucleus, paraventricular nucleus, periventricular and ventromedial hypothalamic nuclei. That study showed some similarities between the action of CGRP in mammalian and non-mammalian vertebrates and shed some light on the mechanisms underlying the observed effects. The general conclusion was that CGRP primarily affected appetite/satiety, reducing food intake.

**Figure 3:**
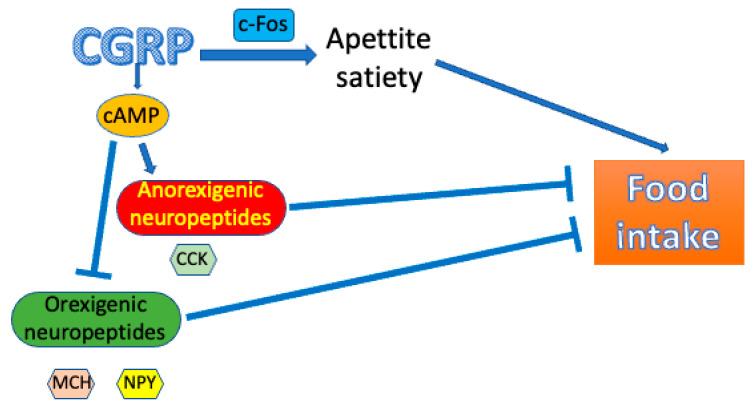
Calcitonin-related gene peptide (CGRP) may exert an anorexigenic effect mediated by c-Fos (Fos proto-oncogene, AP-1 transcription factor subunit) increase through the regulation of appetite and satiety resulting in the modulation of food intake. The regulation of apatite/satiety may be underlined by the upregulation of anorexigenic neuropeptides, including cholecystokinin (CCK) and downregulation of orexigenic neuropeptides, including neuropeptide Y (NPY) and melanin-concentrating hormone (MCH). These effects can be mediated by hypothalamic 3′,5′-cyclic adenosine monophosphate (cAMP).

Mechanisms underlying the anorexigenic potential of CGRP were investigated in rats [79]. Intraperitoneal injection of this neuropeptide resulted in a decline in food intake and an increase in the levels of hypothalamic 3′,5′-cyclic adenosine monophosphate (cAMP) and plasma glucagon, as well as a decrease in insulin level. CGRP injection induced downregulation of the neuropeptide Y (NPY) and melanin-concentrating hormone (MCH) genes, but the cholecystokinin (CCK) gene was upregulated. In these animals, food intake was negatively correlated with CCK mRNA, cAMP and glucagon levels. These results suggest that cAMP acting as the second messenger may play an important role in the anorexigenic effects of CGRP. Therefore, CGRP may induce anorexigenic neuropeptides, such as CCK and/or inhibit orexigenic neuropeptides, such as NPY and MCH, resulting in suppression of food intake, functionally coupled to cAMP (Figure 3).

Anorexigenic behavior in rodents and primates is regulated by the lateral parabrachial nucleus, which is a duct for visceral signals from the caudal hindbrain to forebrain areas linked with appetite control [80,81]. It was identified as a subset of neurons placed in the external lateral parabrachial nucleus (PBel) that expressed CGRP and suppressed feeding and mediated taste aversion when activated by illness mimetics [82]. However, even in normal mice, functional inactivation of PBel CGRP neurons increased meal size and rendered animals insensitive to the anorexigenic effects of meal-related satiety peptides [7]. Furthermore, CGRP neurons are directly innervated by orexigenic hypothalamic agouti-related protein (AgRP) neurons and may play a role in the control of meal termination and feeding provoked by AgRP neurons, controlling feeding behavior.

Although those studies were conducted in rats, there are fundamental regulatory mechanisms that have exerted strong evolutionary pressure [83]. As a consequence, most of the regulatory mechanisms have been conserved from fish to mammals. These mechanisms are underlined by the action of various neuropeptides, including proopiomelanocortin (POMC), NPY, AgRP, cocaine- and amphetamine-regulated transcript (CART), orexin, CCK and MCH. Assorted hormones are also involved—they are insulin, leptin, ghrelin, and glucagon-like peptide 1 (GLP-1).

## 6. CGRP and Metabolic Disorders

Although the cause of obesity is a long-term energy imbalance featured by increased energy intake and reduced energy expenditure, and so it can be avoided by increased physical activity, and dietary nutrients may play an important role in obesity pathogenesis [84].

It is generally accepted that migraine and obesity are related to each other as obesity exacerbates migraine by increasing the risk of the disease and higher frequency and severity of headaches (reviewed in [85]). Although a precise mechanism beyond this association is not known, CGRP may play an important role, as CGRP and substance P (SP) are common inflammatory mediators for migraine and obesity. CGRP level is increased in obese individuals as compared with normal-weight persons, and it further increases after a high-fat meal [86]. Furthermore, the administration of CGRP stimulated fat accumulation in obese animals, and an increase in CGRP level was observed prior to the onset of obesity [85,86,87,88]. On the other hand, CGRP^−/−^ mice were characterized by higher core temperatures, increased energy expenditures, and a relative daytime (nonactive) depression in respiratory quotients, suggesting an increased β-oxidation [88]. In response to fat feeding, these animals were protected against diet-induced obesity with an attenuated body weight gain and a reduction in adiposity. CGRP^−/−^ mice showed improved glucose handling and insulin sensitivity. These results clearly showed the role of CGRP as a mediator of energy metabolism and a target in obesity-reducing treatment.

Type 2 diabetes mellitus (T2DM) occurs more frequently in obese individuals than in persons with normal weight and is associated with impairment in insulin secretion and activity (reviewed in [89]). Although obesity is considered a risk factor in many diseases, including migraine, the mechanisms underlying its involvement in the pathogenesis of these diseases are poorly known. Studies on the association of migraine headaches prevalence and triggers with T2DM did not result in unequivocal outcomes, but some associations between specific characteristics of migraine and T2DM were established [89,90,91]. However, these associations suggest that T2DM, as well as T1DM, may be both a protective and a risk factor in migraine [91]. These facts can be considered within the context of migraine metabolic comorbidities associated with the patient’s lifestyle and genetic constitution.

It was proposed that the early development of insulin resistance, impaired glucose tolerance and T2DM could be modulated by the increased activity of sensory nerves in Zucker diabetic fatty rats [87,92]. Small, unmyelinated sensory nerves can be bound and inactivated by capsaicin, which possibly exerts its action through reduced levels of CGRP, which was shown to induce insulin resistance and inhibit insulin secretion [93]. The general conclusion from these studies was that the increased activity of sensory nerves, associated with increased CGRP release from these nerves, preceded the development of obesity.

It was postulated that CGRP might affect adipocytes to promote lipid utilization and pancreatic β-cells to modulate insulin secretion [94]. Analysis of pancreatic islets with CGRP-specific monoclonal antibodies in mouse models of diabetes and diet-induced obesity showed that CGRP blocked glucose-stimulated insulin secretion and reduced the expression of the insulin-2 gene. Recombinant CGRP reduced glycolytic capacity and fatty acid oxidation in primary white adipocytes. Therefore, circulating CGRP plays an important role in energy metabolism, modulating thermogenic pathways in adipose tissue pancreatic β-cell dependent insulin secretion. Consequently, a decrease in circulating levels of CGRP with monoclonal therapy has therapeutic potential for T2DM in obese mice.

The studies on the role of CGRP and CGRP-related therapy in obese and diabetic animals were translated into two randomized, double-blind, placebo-controlled trials to assess the safety and metabolic effects of eptinezumab, an anti-CGRP antibody, in non-migraine overweight/obese patients and patients with T1DM [95]. No changes in insulin sensitivity between the eptinezumab and placebo groups were observed in T1DM patients. Eptinezumab was well tolerated by all enrolled individuals. In conclusion, eptinezumab was not linked with harmful metabolic effects in overweight/obese and T1DM patients, supporting its use in such patients with migraine.

## 7. Dietary Nutrients May Reduce Gastrointestinal Functional Disorders as Unwanted Side Effects of Anti-CGRP Treatment

Migraine patients are at a higher risk of GI disorders, and this relationship is bidirectional, in agreement with the conception of the gut-brain-microbiota axis [96]. The prevalence of GI diseases was also higher in patients with medications for migraine, both for preventive and acute treatment. The most prevalent GI comorbidities of migraine are gastrointestinal reflux, nausea and vomiting, cyclical vomiting syndrome, abdominal migraine, celiac disease and non-celiac gluten sensitivity, functional diarrhea, functional constipation, and irritable bowel syndrome (reviewed in [97]). The exact mechanisms underlying these associations are not exactly known, but CGRP is a prime candidate to be involved as it is important for many physiological and pathological phenomena and effects in the GI tract (reviewed in [98]).

CGRP and its receptor were localized in enteric neurons in various human GI segments [48,75]. In the stomach, nerve bundles in the myenteric plexus and nerve fibers throughout the circular and longitudinal muscle displayed a prominent expression of CLR. In the proximal colon and ileum, CLR was found in nerve varicosities of the myenteric plexus and surrounding submucosal neurons. Interestingly, CGRP-expressing fibers did not co-localize but were near CLR. However, CLR and RAMP1, the two subunits of the functional CGRP receptor, were localized in the myenteric plexus, where they may form functional cell-surface receptors. Intermedin (IMD), another member of the calcitonin peptide family, was also found close to CLR and, like CGRP, did not co-localize with either CLR or RAMP1 receptors [48,75]. Thus, CGRP and IMD appear to be released locally, where they can mediate the effect on their receptors regulating diverse functions such as inflammation, pain and motility.

Constipation is frequently mentioned as a gut-related adverse effect of anti-CGRP migraine therapeutics as the post-approval real-world surveys showed that it might occur in more than 50% of patients treated with erenumab, an antibody targeting the CGRP receptor, fremanezumab, or galcanezumab, antibodies targeting CGRP (reviewed in [99]). CGRP is a major messenger of enteric sensory neurons, which activate both ascending excitatory and descending inhibitory neuronal pathways that facilitate peristaltic activity. CGRP stimulates ion and water secretion into the intestinal lumen. This stimulatory activity of CGRP might result in diarrhea and other unwanted gut-related effects. It was confirmed in a study in which 2 hours of CGRP infusion was associated with rumbling, stomach pain, nausea, diarrhea, and an urge to defecate were the most commonly experienced GI side effects [100]. These symptoms were not antagonized by sumatriptan. These studies suggested that constipation might be a side effect of anti-CGRP therapy. It was concluded that the constipation provoked by antibodies targeting CGRP or its receptor resulted from interference with the physiological functions of CGRP in the small and large intestines, including the maintenance of peristaltic motor activity, ion and water secretion and intestinal transit [99].

FDA Adverse Event Reporting System (FAERS) shows that the prevalence of adverse, GI-related syndromes associated with the administration of CGRP-based antibodies ranges from 0.3% (diarrhea) to 7.6% (nausea) (reviewed in [98]). Gepants also show the highest prevalence (10.4%) of nausea among GI-related syndromes. In general, GI-related functional syndromes may be considered the most serious side effects of anti-CGRP therapy.

Recent randomized controlled trials and real-life studies provided important information on GI homeostasis and anti-CGRP therapy. The multicenter prospective cohort GARLIT real-life study enrolled patients with high-frequency episodic migraine and chronic migraine [101,102]. It was shown that galcanezumab, a humanized monoclonal antibody against CGRP, was effective and well-tolerated in the 1-year term, but persistent responders were less likely to have a higher BMI. It was concluded that lower BMI in the first month of the treatment with galcanezumab was among the factors predicting a persistent response to this drug in migraine patients. Another anti-CGRP antibody, eptinezumab, showed a clinically relevant reduction from baseline in mean monthly migraine days compared with placebo in adults with episodic (the PROMISE-1 study) or chronic (PROMISE-2) migraine [103]. Although the 100- and 300-mg doses did not exceed a 10% separation from placebo on the ≥50% migraine responder rate for the obesity class II (BMI 35 to <40 kg/m^2^) patients, and the 100-mg dose did not exceed a 10% separation for certain subpopulations of patients (obesity classes I (30 to <35 kg/m^2^) and II, patients with migraine diagnosed between 20 and 30 years of age, and patients diagnosed less than 9 years), the fraction of migraineurs with ≥50% migraine responder rate was higher for eptinezumab- than placebo-treated patients. Therefore, these studies point to BMI as a factor that can modulate anti-CGRP therapy, but this issue should be addressed in further research also in the context of nutrition. An explorative, prospective, questionnaire-based study showed that most chronic migraine patients treated with CGRP antibodies displayed complete prevention of migraine symptoms without indication of initial onset followed by attack abortion [104]. About one-fifth of patients reported an increase in appetite, and a similar number displayed an increase in weight. A 3-month observational, longitudinal, cohort, multicenter, Italian real-life study showed that galcanezumab showed that most patients displaying a ≥50% migraine responder rate showed a lower BMI [105]. Furthermore, at baseline, over 80% of patients presented medication overuse, and most of these no longer showed MO consistently during the 3 months of anti-CGRP therapy. These patients less frequently suffered from gastrointestinal comorbidity (*p* = 0.007). Altogether, these studies show that gastrointestinal homeostasis may be associated with the efficiency of anti-CGRP therapy. Therefore, nutrition may play a role in this fundamental anti-migraine therapy.

In summary, CGRP is an important element of the gut-brain axis, and its modulation may lead to GI functional disorders. Such disorders are reported to be side effects of anti-CGRP therapy in migraine. However, this association may be featured by several factors, including intake of dietary nutrients, as it may be involved in both migraine and GI tract disorders. Dietary nutrients improving functions of the GI, in particular preventing or inhibiting diarrhea and constipation as well as nausea, may be useful in reducing unwanted side effects of anti-CGRP migraine therapy.

## 8. Concluding Remarks and Perspectives

Dietary nutrients are of concern to migraine patients as some of them are migraine triggers and may exacerbate migraine headaches. On the other hand, CGRP, a central neuropeptide in migraine pathogenesis, is sensitive to many dietary nutrients. This sensitivity may influence CGRP properties that could contribute to migraine pathogenesis, such as light aversion, neurogenic inflammation, peripheral and central sensitization of nociceptive pathways, cortical spreading depression, and regulation of nitric NO production [45]. Furthermore, CGRP is involved in the regulation of many functions in the GI tract, which can be modulated by dietary nutrients.

Many studies associate the diet with migraine pathogenesis, first of all recommending dietary components to avoid so as not to induce headaches or exacerbate existing ones. Much fewer studies deal with dietary components that are recommended to be added to the standard diet. As with any kind of diet, migraine preventive diets should be adjusted to individual needs and constitutions, understood as the set of genetic/epigenetic and phenotypic features.

The introduction of antibodies against CGRP, along with antibodies and antagonists to its receptor, revolutionized the prevention and treatment of migraine, but several outstanding questions remain [106]. Some of them, especially those on the long-term use of these drugs, will be answered in the future as more information on the efficacy and safety of these drugs is gathered. The main problem with anti-CGRP therapy, the explanation as to why some migraine patients are insensitive to it, is intensively studied, and we do not suggest that the solution to this problem lies in the modulation of the intake of dietary nutrients. However, we tried to show that nutritional aspects of CGRP functioning, including its role in migraine pathogenesis and migraine therapy, are complex and associated with many aspects of the functioning of the gut-brain axis.

Another problem associated with anti-CGRP is the unwanted side effects of the therapy. Generally, such therapy is considered safe, but some patients complain of ailments, mainly from the GI tract [97]. Therefore, the link between dietary nutrients and CGRP is especially important as these nutrients may influence anti-CGRP therapy affecting CGRP and modulating functions of the GI tract. CGRP in the GI tract is expressed by two distinct neuronal populations: extrinsic primary afferent nerve fibers and diverse neurons of the intrinsic enteric nervous system. The anti-CGRP migraine therapeutics can affect CGRP expressed in both these subpopulations. Consequently, the GI tract-related effects of these therapeutics should be specifically attributed to one of these populations or both.

The anorexigenic potential of CGRP is important in any dietary intervention in migraine patients and obviously impedes such intervention. Therefore, dietary nutrients recommended in migraine and in anti-CGRP therapy should be provided in the form of diet supplements rather than natural products. Additionally, obesity and metabolic diseases, including diabetes, that may be influenced by CGRP make this problem even more complex.

The fundamental outstanding question is whether dietary nutrients may be used to improve anti-CGRP migraine therapy making it more effective and/or safe. To address this question, many elements should be taken into consideration, including two fundamental ones. Firstly, dietary nutrients that are migraine triggers should be excluded as such compounds, even if they may improve the mechanistic action of ant-CGRP drugs. Secondly, nutritional strategy in obese and/or diabetic patients in anti-CGRP therapy should be adjusted to the anorexigenic potential of CGRP.

This review does not answer important questions on the use of nutrients in anti-CGRP therapy. Such questions can be addressed when outcomes of clinical trials and results of laboratory studies are available. This review aimed to underline the importance of dietary nutrients in anti-CGRP therapy of migraine and justify the need for further research as the currently available data are scarce.

## Figures and Tables

**Table 1:** Some diets that are reported to have beneficiary effects in migraine prevention and treatment with some exemplary references.

Diet	Feature	Reference
Healthy Eating Plate	Half of the plate is dedicated to fruits and vegetables, a quarter to whole grains, and a quarter to proteins.	[24,25]
Ketogenic diet	A strong restriction of carbohydrates with a higher intake of lipids and proteins.	[26,27,28]
Gluten-free diet	Avoids ingestion of wheat, rye, barley, malt, and their derivatives and, instead, encompasses gluten-free alternatives such as rice, quinoa, corn, and potatoes and food groups that are naturally devoid of gluten such as fruits, vegetables, seafood, meat, legumes, nuts, and most dairy products.	[29,30,31]
Low-calorie diet	Assumes consuming around 1200 to 1500 calories per day	[32]
Low-glycemic index diet	Contains items such as most non-starchy vegetables, beans and legumes, nuts, and most fruits, but not pineapple and watermelon	[33,34]
Mediterranean diet	Emphasis on whole grains, vegetables, legumes, fruits, nuts, olive oil, and moderate animal-based protein, excluding meat	[35,36,37]
Fatty acid diet	Various adjustments to the composition of fatty acids	[38,39]

## Data Availability

Not applicable.
